# A Predictive and an Optimization Mathematical Model for Device Design in Cardiac Pulsed Field Ablation Using Design of Experiments

**DOI:** 10.3390/jcdd10100423

**Published:** 2023-10-11

**Authors:** Eoghan Dunne, Jara M. Baena-Montes, Kevin Donaghey, Cormac Clarke, Marcin J. Kraśny, Bilal Amin, Tony O’Halloran, Leo R. Quinlan, Adnan Elahi, Martin O’Halloran

**Affiliations:** 1 Translational Medical Device Lab (TMD Lab), Lambe Institute for Translational Research, School of Medicine, University of Galway, H91 TK33 Galway, Ireland; 2 Physiology and Cellular Physiology Research Laboratory, CÚRAM SFI Centre for Research in Medical Devices, School of Medicine, Human Biology Building, University of Galway, H91 TK33 Galway, Ireland; 3 AuriGen Medical, GMIT Innovation Hubs, H91 DCH9 Galway, Ireland; 4 Smart Sensors Lab, Lambe Institute for Translational Research, School of Medicine, University of Galway, H91 TK33 Galway, Ireland; 5 Electrical & Electronic Engineering, College of Science and Engineering, University of Galway, H91 TK33 Galway, Ireland

**Keywords:** pulsed field ablation, atrial fibrillation, in vitro, cardiomyocytes, design of experiments

## Abstract

Cardiac catheter ablation (CCA) is a common method used to correct cardiac arrhythmia. Pulsed Field Ablation (PFA) is a recently-adapted CCA technology whose ablation is dependent on electrode and waveform parameters (factors). In this work, the use of the Design of Experiments (DoE) methodology is investigated for the design and optimization of a PFA device. The effects of the four factors (input voltage, electrode spacing, electrode width, and on-time) and their interactions are analyzed. An empirical model is formed to predict and optimize the ablation size responses. Based on the ranges tested, the significant factors were the input voltage, the electrode spacing, and the on time, which is in line with the literature. Two-factor interactions were found to be significant and need to be considered in the model. The resulting empirical model was found to predict ablation sizes with less than 2.1% error in the measured area and was used for optimization. The findings and the strong predictive model developed highlight that the DoE approach can be used to help determine PFA device design, to optimize for certain ablation zone sizes, and to help inform device design to tackle specific cardiac arrhythmias.

## 1. Introduction

Cardiac catheter ablation is a minimally invasive method to treat arrhythmias. The method involves applying energy to the cardiac tissue to form lesions at sites where action potentials are associated with the arrythmia flow [1]. The resulting lesions electrically block the triggers and conduction pathways, helping to treat the arrhythmia [1,2]. The energy has been typically applied using thermal methods such as radiofrequency ablation [3]. However, thermal methods can have complications, including pulmonary vein stenosis and damage to the esophagus, the phenic nerve, and coronary arteries [4]. Pulsed field ablation (also known as irreversible electroporation) is an on-the-rise technology for cardiac catheter ablation. The technology involves the application of short-duration, high-voltage pulses through electrodes that cause increased membrane permeability [5]. If applied with a sufficiently strong electric field and for a sufficiently long on- time, irreversible damage to the cell will occur, ultimately resulting in cell death [5]. The benefits of pulsed field ablation include non-thermal scar tissue formation, quick delivery of the energy, tissue selectivity, and deep lesion formation, as well as a low risk of pulmonary vein stenosis, esophageal damage, and collateral coronary damage [2,6,7].

**Figure 1:**
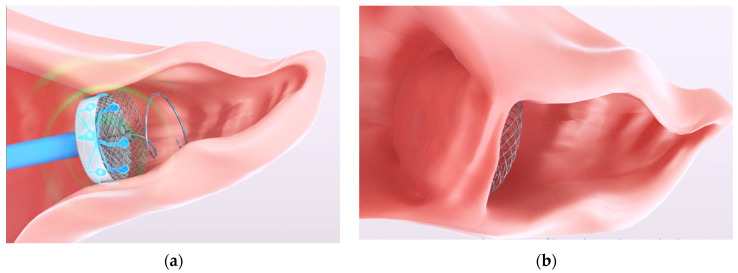
Illustration of a new solution being developed by AuriGen Medical (aurigenmedical.com) to help treat PeAF. The method involves combining ablation and occlusion of the left atrial appendage in order to treat AF and prevent clots that may have formed within the appendage from escaping and causing further problems, such as stroke [8]. (**a**) Ablation after inserting the occlude and (**b**) the occluder sealing the left atrial appendage after endothelization.

Using pulsed field ablation, a solution is being developed by AuriGen Medical (aurigenmedical.com (accessed on 3 August 2023)) to target the arrhythmia of atrial fibrillation (specially, longstanding persistent atrial fibrillation) by combining the occlusion of the left atrial appendage with ablation in one single procedure. Hence, helping to correct atrial fibrillation by electrical isolation of the left atrial appendage as well as occluding the region to prevent dislodgement of any thrombus accumulation in the appendage (and potential of subsequent stroke [8]). This concept can be seen in Figure 1. During the design of this device, prediction and optimization of the ablation sizes are required. The pulsed field ablation depends on the electrode size and spacing as well as the waveform used to deliver the energy. These factors need to be considered when devising a predictive mathematical model.

In this work, the objective is to create an empirical mathematical model for prediction and optimization of the ablation size in an in vitro cardiomyocyte model based on the design constraints of the concept in Figure 1. The mathematical model created needs to consider the waveform as well as the physical features of the device in order to predict or optimize the ablation size. Therefore, the parameters (or factors, as referred to in this paper) of interest are the input voltage, the on-time of the treatment, the electrode size (controlled by the electrode width), and the electrode spacing.

One-factor-at-a-time (single factor) experiments are often performed, i.e., where one factor is varied to see how the response changes while the other factors are held constant [9]. However, these experiments do not consider the interactions between the factors [10], e.g., the combination of the electrode width and the input voltage on the ablation size. Design of Experiments (DoE) is a methodology that takes into account of how the factors and the interactions between the factors affect the response (dependent variable) and helps determine mathematical relationships that can be used to predict or optimize the response [9]. The core benefit of the method is that the method focuses on running the minimum number of experimental runs to maximize the knowledge that can be gathered from the experiment [10].

DoE (including Response Surface Designs) has been previously used in the electroporation field. The tool has been used in optimizing designs to determine optimal electrode and waveform factors in an in silico liver model and to avoid thermal damage in an in silico liver model [11,12], in optimizing food processing and industrial production [13,14], and in optimizing electroporation for gene delivery [15]. Therefore, DoE is used in this study to help with device design and optimization for the ablation of cardiomyocytes in vitro. The resultant mathematical models are then used to optimize for conditions of interest and to verify the model is performing beyond the training data.

The rest of the manuscript is divided into the description of the methods in Section 2; the results and discussion in Section 3; and finally, the paper is concluded in Section 4.

## 2. Materials and Methods

In this section, the overall experiment design is presented. Then, the individual parts of the experiment are discussed, including cell culture preparation, probe design and ablation setup, and ablation imaging and postprocessing.

### 2.1. Experimental Design with Design of Experiments

In this work, a full factorial design was used. A full-factorial design is one where every combination of the factors is considered. For a four-factor experiment, with each factor having two endpoints to represent the desired range (two levels), the number of runs would be sixteen (2^4^ runs). To increase the power of the experiment, the sixteen runs would be replicated several times. In this experiment, the full 16 runs are repeated twice (two design replicates). Within each design replicate, the order of the runs is randomized. To allow for ablation, imaging, and analysis, the experiment was divided into 4 blocks. A block was executed each day, with the full experiment conducted over four days. The final design can be summarized as a two-level, four-factor, two-design replicates, blocked, randomized full-factorial design.

The full-factorial design is based on assuming linear relationships between both the factors and the factor interactions with the response. Center points allow for the detection of curvature [9]. However, center points were not added for this experiment under the assumption that we expected linear behavior. If this assumption was found to be invalid based on indications from a poor fit of the model, DoE allows for the addition of center points after the experiment if curvature need to be confirmed. Further, the design could be expanded to a response surface design to address the non-linear relationship, if needed.

Minitab^®^ (Minitab LLC, minitab.com, version 19, Coventry, United Kingdom) was used to generate the DoE design. The levels used for each of the four factors are given in Table 1, and the run order is given in Supplementary Material, Table S1.

The factor of the number of ablation repeats was used as a proxy for the on-time. The on-time can be altered by adjusting the pulse width, the number of pulses, the number of bursts, or the number of repetitions of the waveform used. Due to limitations with the generator employed, there was no inter-burst recharging. Hence, the waveform was repeated several times (number of ablation repeats) to overcome this obstacle.

The limits were determined by preliminary experimentation or by design requirements. For example, the maximum value of input voltage was decided upon as 1300 V, as higher voltages showed either excessive bubble formation or spark formation in controlled experiments. The lower value of 1000 V was used as lower voltage ranges were not of interest in the device design as higher voltages lead to higher electric field strength and hence, larger ablations. The values of the number of ablation repeats were decided based on previous results that indicated a minimum of four repeats were needed to obtain a sufficiently large enough ablated area (nearly bridged or bridged). The electrode width and electrode spacing were design constraints for the future implant design. While the ranges were small, in particular the electrode width (a range of 0.3 mm), the ranges were chosen to enable the implant to be deployed.

### 2.2. In Vitro Cell Culture

The human cardiomyocyte cell line AC16 (Merck Life Science Limited, Arklow, Co., Wicklow, Ireland) was used. The cells were cultured in a growth media consisting of Dulbecco’s Modified Eagle’s Medium/Ham’s Nutrient Mixture F-12 (DMEM/F-12) (Merck Life Science Limited, Arklow, Co., Wicklow, Ireland), supplemented with 12.5% Fetal Bovine Serum (FBS) (Life Technologies, Burlington, ON, Canada), 1% Penicillin/Streptomycin (Merck Life Science Limited, Arklow, Co., Wicklow, Ireland), and 2 mM L-Glutamine (Merck Life Science Limited, Arklow, Co., Wicklow, Ireland). The cultures were maintained at 37 °C in a humidified atmosphere with 5% CO_2_. The experimental treatment cells were seeded at a density of 7.5 × 10^5^ on precoated (0.1% gelatin) 6-well plates.

### 2.3. The Ablation Setup and the Ablation Probe

**Figure 2:**
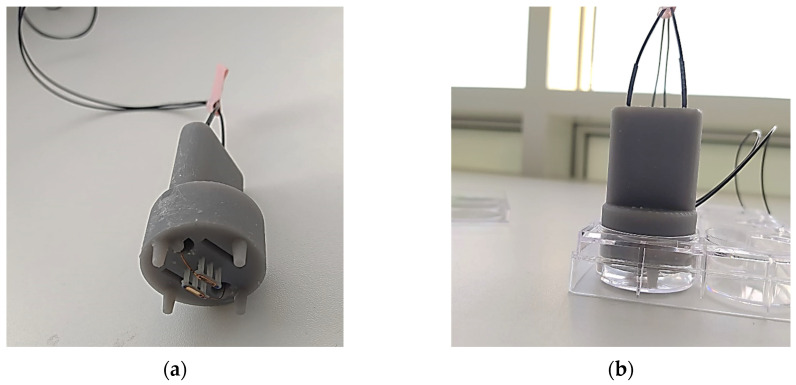
Alation probe design and fitting. (**a**) A custom-printed probe that represents the intended electrode design and spacing for the implant; and (**b**) the probe set up within the well for ablation.

After incubation for 24 h, the media was removed and substituted with 5 mL of 0.3% sodium chloride. This solution was used to mimic the conductivity of blood. A 0.3% sodium chloride solution is approximately 5.28 mS/cm [16], which fits the human blood measurements by Istuk et al. [17] at around 37 °C. Samples were ablated in the culture well plate using custom 3D-printed probes. The electrodes were fabricated using copper and had a height of 4.5 mm and filled corners of 0.6 mm radii. The electrodes were connected to the wave generator. The electrodes were placed approximately 1 mm above the cell layer using the four plastic legs of the probe. This height above the cell layer was used to avoid the electrodes being in direct contact with the cell layer. The probe is shown in Figure 2.

The remaining waveform parameters were fixed. Due to proprietary reasons, these fixed waveform parameters are not given.

### 2.4. Ablation Imaging and Postprocessing

**Figure 3:**
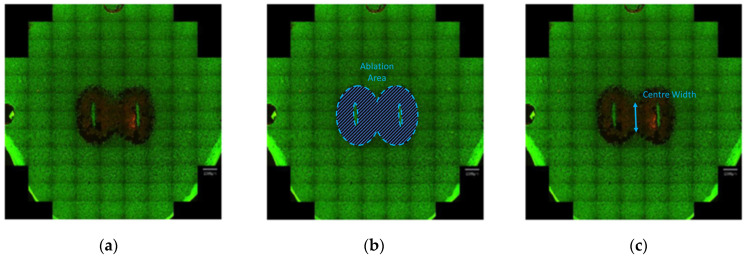
Illustration of the measured responses for the DoE: (**a**) the reference fluorescent scanning image; (**b**) the ablation area; (**c**) the center width. These responses are relevant to the design of the ablation, as the area captures the ablated region in 2D, and the center width is used to help determine if the ablation is bridged or not, as well as to help control the size of the ablation between the electrodes.

Post-ablation, the cell monolayers were incubated with 1.5 µM Calcein-AM and 3 µM Propidium Iodide (PI) (30 min, 37 °C) (Sigma-Aldrich), facilitating the identification of live and dead cells, respectively. The extent of cell death in the monolayer was assessed two hours post-ablation using the live/dead assay by imaging monolayers on the EVOS M7000 Imaging System microscope (Life Technologies Corporation, Bothell, Washington, DC, USA). Captured images were further analyzed using National Institutes of Health ImageJ [18] to determine the ablation area and center width. Scaled images were split into color channels. The green channel was chosen to help determine the ablation area (the ablation area being more visible than the area in ‘black’ or with no green-positive cells). The image threshold was established such that it detected the ablation area only. After the application of the threshold, the image was transformed into a binary image, and the Analyze Particles tool was applied. The ablated area and center width were measured (Figure 3).

## 3. Results and Discussion

In this section, the results are discussed in the order of the ablation images and measurements, the DoE analysis, the confirmation analysis of the DoE, and finally, an optimization to determine suitable parameters for electrical isolation by a given definition.

### 3.1. Ablation Images and Measurements

**Figure 4:**
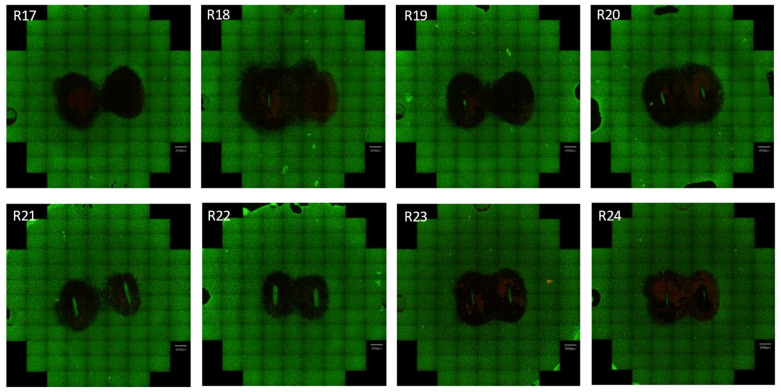
Images of the 2D cell ablations in block 1 are labeled with corresponding run numbers. The ablation region can be seen in the center of the image and is marked by the black color. Live cells are marked by a green color. In this block, a fully bridged ablation was obtained for each run. Having a fully bridged ablation is important to electrically isolate the region for the intended application of ablating the left atrial appendage.

Figure 4 shows the captured fluorescent scanning images of the ablated well for one of the four blocks from the DoE experiment. The remainder of the images can be found in the Supplementary Material, Figure S1. In 31 of 32 ablations, a fully bridged ablation was achieved (measured center width > 0). The range of the ablation areas varied from 63.43 mm^2^ to 134.89 mm^2^. The full list of measurements made is given in the Supplementary Material, Table S2, along with the run order.

In some images, regions outside the main ablation show regions that may be misinterpreted as ablations, e.g., runs 11 and 27. However, these regions were determined to be associated with cell detachments. Additionally, for certain runs, an unablated region occurs below where the electrode was placed. The lack of ablation is assumed to be due to the close contact of the electrodes with the cells underneath the electrode and insufficient voltage or insufficient on-time.

The data measured from the ablated images was then transferred to DoE software for analysis, as is discussed in the next subsection.

### 3.2. Design and Experiments Analysis and Discussion

The responses (dependent variables) of interest in this work were the ablation area and the center width measurement. The center width measurements were taken to capture a bridged ablation and to allow the option to optimize along with or constrain this response when optimizing the ablation area.

**Figure 5:**
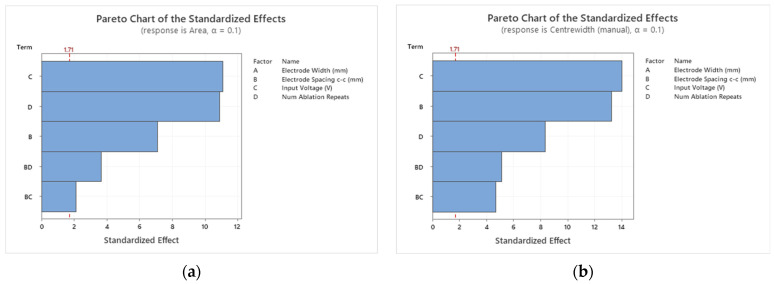
The Pareto charts for the ablation area (**a**) and the center width responses (**b**) indicate what factors have the largest effects on the response. All factors and interactions shown are significant, as the model was fully reduced by backward elimination. For the ablation area, the input voltage has the largest effect. The two-factor interactions have the least. The input voltage has the largest effect on the ablation area as well as the center width response. The two significant two-factor interactions have the least effect.

A DoE analysis was performed with the constraint of up to two-factor interactions. Three-factor interactions or higher are considered rare [9]. Backward elimination at a significance level of 0.1 was used to form a fully reduced model, i.e., removing insignificant terms from the prediction or optimization model. The significance level of 0.1 allowed for finding all potentially helpful factors for the ablation. Then, analysis by the team could be used to determine if the remaining factors with *p*-values between 0.05 and 0.1 do indeed impact the ablation. Figure 5 shows the Pareto charts for both responses.

For both ablation area and center width, the significant factors were the input voltage, the electrode spacing, and the number of ablation repeats. The two-factor interaction of electrode spacing and input voltage, the two-factor interaction of electrode spacing, and the number of ablation repeats were also significant. Each of these factors and two-factor interactions had a *p*-value of less than 0.05. The only factor not to be found significant in this study was the electrode width. The insignificance of the electrode width may be attributed to the narrow range of the electrode width parameter (1.2–1.5 mm, i.e., a difference of 0.3 mm) used in the experiments. If a wider range was of interest and was tested, the results may have differed for the electrode width. However, the design constraint is based on being able to deliver and deploy electrodes attached to an occluder (as shown in Figure 1) through a catheter and into the left atrial appendage.

**Figure 6:**
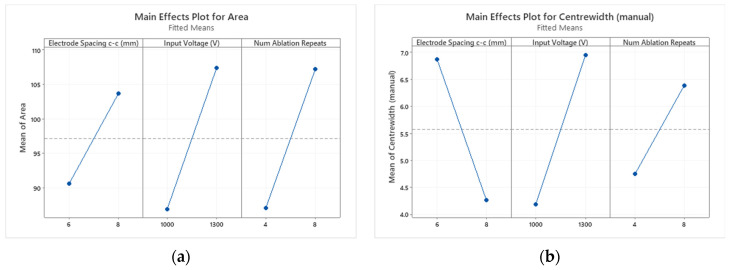
Main effects plot for (**a**) the ablation area and (**b**) the center width responses. Increasing each significant factor causes the ablation area to increase. Increasing either the input voltage or the number of ablation repeats causes the center width to increase. However, increasing electrode spacing causes the center width to decrease.

For both responses, the input voltage has the largest effect (similar to the finding by Yang et al. [11] for in silico ablation of the liver). The two-factor interactions have the least effect on both responses. The two Pareto charts differed based on the order of the electrode spacing and the number of ablation repeats. The electrode spacing has more of an effect on the center width response than the number of ablation repeats. This finding makes sense, as by increasing the electrode spacing while keeping all other factors constant, it would be expected that the center width would reduce. This hypothesis is confirmed in the main effects plot, which can be seen for the ablation area and center width responses in Figure 6, respectively.

The ablation area was observed to increase with an increase in each factor. For the center width, the same pattern was observed except for the electrode spacing. As the electrode spacing increased, the center width decreased. Intuitively, these findings make sense.

#### 3.2.1. Observations with Large Residuals (Greater Than Two Standard Deviations)

It is expected to have roughly 5% of the observations be unusual observations (large residuals), since unusual observations are defined by the software as outside two standard deviations [19]. In this experiment, 1.6 runs (two whole runs) would be classified as unusual observations for 32 runs, altogether.

Two runs were flagged as unusual for the ablation area response, and one run was flagged for the center width response. In the case of the center width response, measurement error may have been possible due to difficulty in determining the center width measurement when cells are within the middle region of the ablation. However, this is a limitation of the measurement approach.

#### 3.2.2. The Developed Empirical Mathematical Model

DoE is analyzed using analysis of variance (ANOVA), where the variance is partitioned into within- and between-factor contributions. A global average is first calculated, followed by an evaluation of the size of the individual factor and interaction effects. The ANOVA analysis of a factorial DoE is based on “coded” factors. Coded means that numerical input factors are calculated on a −1 to +1 scale for the lowest and highest values, respectively. If there are categorical factors, they are also treated on a −1 and +1 basis, but there is no allowance for intermediate values. The reason coded values are used is to allow for the difference in units and scales between the factors. For example, there is no direct way to compare input voltage to electrode spacing. The use of coded factors provides a more fundamental understanding of how those factors contribute to the mathematical model (coded models can be found in the Supplementary Material). Further details on the mathematical equation formation can be found in Montgomery 2013 [9]. After the formation of the coded mathematical model, the mathematical model can be transformed into an equation of the original units (i.e., uncoded) for prediction. These models below are for both the ablation area and the center width.

The mathematical model for the ablation area is:

(1) $$Ablation\;Area=118.4-18.50\;{Dist}_{elec}\;-0.0231\;V_{in}-6.74\;N_{repeats}\;+0.01301\;{Dist}_{elec}\;V_{in}+1.678\;{Dist}_{elec}\;N_{repeats}$$

where ${Dist}_{elec}\;$ is the electrode spacing c-c (in millimeters), $V_{in}$ is the input voltage (in volts), and $N_{repeats}$ is the number of ablation repeats.

The mathematical model for the center width is:

(2) $$Center\;Width=37.14-6.371\;{Dist}_{elec}-0.01243\;V_{in}-1.357\;N_{repeats}\;+0.003089\;{Dist}_{elec}\;V_{in\;}+0.2525\;{Dist}_{elec}\;N_{repeats}$$

For the developed empirical models, the R-sq(adj) and the R-sq(pred) were found to agree with each other within 20%, indicating good predictive models for both the ablation area and center width responses [20], p. 105. These models can be used to help determine suitable design inputs to optimize or target certain ablation sizes.

### 3.3. Confirmation Runs of the Empirical Model

Confirmation runs were used to verify the ablation area model (the model of most interest) would work for new predictions outside the training data as well as to verify the model could be used beyond the experiment. Four runs were predicted with the following specifications:

- An ablation area of approximately 97 mm^2^ with the constraints of 8 mm electrode spacing and an input voltage of 1000 V;
- An ablation area of approximately 97 mm^2^ with the constraints of 8 mm electrode spacing and an input voltage of 1300 V;
- An ablation area of approximately 80 mm^2^ with the constraints of 8 mm electrode spacing;
- An ablation area of approximately 110 mm^2^ with the constraints of 8 mm electrode spacing.

The 1.5 mm electrode width was also a criterion. However, this criterion did not influence the model, as the model was fully reduced to only include the significant factors and the significant two-factor interactions. A limitation of this work was that the confirmation was not performed for 6 mm electrode spacing or the center width response. Future experimental work can be performed to verify these conditions.

The confirmation runs that were outputted are given in Table 2. It should be noted that the values of the number of ablation repeats were rounded as the number is a natural number. The expected areas, the areas measured from the ablation runs, and the 98.75% prediction interval are also given in Table 2. The 98.75% prediction interval was calculated as four confirmation runs were used to assess the model. This prediction interval was determined using the Bonferroni correction [9], as multiple confirmation runs were being used to confirm the DoE.

Each confirmation run agreed within 3 mm^2^ of the predicted area, which is considered a small error (<2.1%). The values were within the predicted interval. Hence, the findings indicated a reliable model for predicting the ablation area for the factors: electrode spacing; input voltage; number of ablation repeats; and electrode width.

### 3.4. Optimizing Ablation Area for Electrical Isolation

With pulmonary vein isolation, the primary method of assessing whether the treatment has been successful is to measure the potential before and after ablation (scar tissue is classified when the bipolar voltage of the measured tissue is below 0.5 mV [21]).

To ensure electrical isolation of the region, electrical signals must not flow into the left atrial appendage. In this work, the surface area is primarily considered as a monolayer of cardiomyocytes in an in vitro model employed in the study. For this application, a center width equal to the height of the electrodes (4.5 mm) is considered a sufficient thickness for a bridged ablation. Further testing in vivo can be performed to verify that the region is isolated by measuring the electric potential. Further experimentation can also be performed to determine if the lesion size shrinks in follow-ups, one or multiple months after treatment. This information may determine the tolerance of the center width of the ablation.

Using the target of 4.5 mm for the center width and the constraint of 8 mm for the electrode spacing, the ablation size was maximized. The factor values were found to be 1060.97 V and eight ablation repeats, which would have resulted in an ablation size of 109.84 mm^2^ (95% CI: 105.72 mm^2^ to 113.97 mm^2^; 95% PI: 98.32 mm^2^ to 121.36 mm^2^).

### 3.5. Limitations of the Study and Future Work

In this study, an in vitro model (a planer layer of cardiomyocytes with a saline layer above to mimic blood) was employed to assess the effect of ablation parameters using a Design of Experiments (DoE) approach as well as to develop a predictive and an optimization mathematical model. The in vitro model employed has limitations, including a lack of 3D depth that would be found in vivo, the potential of cells detaching from the plastic cell culture plate base, and the inability to assess contact force or angle. However, in vitro models have been previously used for assessment of pulsed field ablation in several studies [22,23,24], despite the outlined limitations. In this study, the model was purposeful as the model captured cardiac-related cell types, characterized the general response to applied electrical fields, and facilitated the initial development of the electrodes and rapid testing of different pulse protocols. The model also enabled cost-effective and accurate analysis of the ablated area (in this case, via fluorescent microscopy). Further work is needed to determine the differences in pulsed field ablation in cell culture and in whole tissue models. However, this question is beyond the scope of this work.

Further, for the specific application of electrical isolation of the left atrial appendage, depth needs to be investigated and optimized for as the depth fluctuates around the left atrial appendage [25]. As part of this future work, electro-tissue contact also needs to be accounted for. A recent study has highlighted that while PFA can cause lesions without direct contact, direct contact of the electrodes on the tissue is needed to maximize the ablation depth [26]. This future investigation of electro-tissue contact may be performed by simulation and/or using a reanimated heart model, similar to the one in Howard et al. 2022 [26].

Additionally, in this study, the work was performed experimentally to capture the resulting ablation from the overall system, including the custom generator characteristics. Numerical modeling could also have been performed to further enhance the work. For example, in determining the electric field strengths of the ablation and in comparing how the results of the DoE performed in simulation compare to the experimental results. Future work will further analyze the data numerically.

## 4. Conclusions

In this work, the relationships of the factors and their interactions with the ablation responses were determined. An empirical model was created for the prediction and optimization of ablation sizes in an in vitro cardiomyocyte model.

Three factors and two sets of two-factor interactions were found to significantly affect the ablation responses. The significant factors were input voltage, electrode spacing, and the number of ablation repeats. The factor with the largest effect for both the ablation area and center width responses was the input voltage, like what was found in the literature previously in the case of liver ablation [11]. Surprisingly, the electrode width was not found to be significant in this study. This finding may be due to the narrow range required for the electrode width in the device under design.

The two-factor interaction of electrode spacing and input voltage and the two-factor interaction of electrode spacing and the number of ablation repeats were found to be statistically significant and need to be taken into account in the model.

Four confirmation runs were performed to confirm the model. All four runs were measured areas agreeing within 2.1% of the predicted areas, and the measured areas were within the expected prediction interval. Further, the factor values were found to be 1060.97 V and eight ablation repeats that would result in an ablation size of 109.84 mm^2^ (95% PI: 98.32 mm^2^ to 121.36 mm^2^) to meet the criteria of isolating the region of interest, as defined and used in this study.

Further work is needed to determine if this model can be used beyond in vitro device optimization. However, the strong predictive model will be used to help determine device/waveform settings to target certain ablation sizes and inform device design in cardiac catheter ablation.

## Figures and Tables

**Table 1:** The factors used in the experiment along with their corresponding ranges.

Individual Factor	Description	Low	High
Input Voltage (V)	Voltage level of the waveform	1000	1300
Number of Waveform/Ablation Repeats	Proxy for the on time	4	8
Electrode Spacing c-c (mm)	Center-to-center distance between the electrodes	6	8
Electrode Width (mm)	Edge-to-edge distance of the electrode	1.2	1.5

**Table 2:** The confirmation runs are used to assess the model along with the results. All values are rounded to two decimal places.

Electrode Width (mm)	Electrode Spacing (mm)	Input Voltage (V)	Number of Ablation Repeats	Area Expected (mm^2^)	Area Measured (mm^2^)	98.75% Prediction Interval (mm^2^)
1.5	8	1000	7	98.22	98.05	(83.17, 113.27)
1.5	8	1300	4	102.45	100.35	(87.10, 117.81)
1.5	8	1000	4	78.16	77.15	(62.80, 93.51)
1.5	8	1150	7	110.37	108.28	(95.73, 125.00)

## Data Availability

All data are available in the manuscript with regard to the DoE and the experimental. The only exception is the specific waveform parameters for the fixed parameters used in this work. The parameters are not given due to proprietary reasons.

## Supplementary Materials

The following supporting information can be downloaded at: https://www.mdpi.com/article/10.3390/jcdd10100423/s1, The supplementary material includes information on the run order, ablated cell images and data analysis files exported from Minitab. Table S1: Run order for the DoE performed; Figure S1: Ablated cells in well for each of the runs in Blocks 2, 3, and 4; Table S2: The Measured Areas and Center Widths Per Run; Section S4: Minitab Regression Analysis for Ablation Area and Center Width; Section S5: Confirmation Run Optimisation–Minitab Analysis; Section S6: Confirmation Run Analysis (Prediction for Area @ 98.75%)–Minitab Analysis; Section S7: Electrical Isolation Optimisation–Minitab Analysis
