# Supplementary material for: A Predictive and an Optimization Mathematical Model for Device Design in Cardiac Pulsed Field Ablation Using Design of Experiments
Source: J Cardiovasc Dev Dis. 2023 Oct 11;10(10):423. doi: 10.3390/jcdd10100423 (PMC10607717; doi:10.3390/jcdd10100423)
Supplement: Supplementary file 1 [file jcdd-10-00423-s001.zip › jcdd-2567519-supplementary.pdf]

Supplementary Material

A Predictive and an Optimization Mathematical Model for Device Design in Cardiac Pulsed Field Ablation using Design of Experiments

Eoghan Dunne, Jara M. Baena-Montes, Kevin Donaghey, Cormac Clarke, Marcin J. Kraśny, Bilal Amin, Tony O'Halloran, Leo R. Quinlan, Adnan Elahi and Martin O'Halloran

S1: DoE Run Order

Table S1: Run order for the DoE performed.

Std Order	Run Order	Center Point	Blocks	Electrode Width (mm)	Electrode Spacing c-c (mm)	Input Voltage (V)	Number of Ablation Repeats
17	1	1	3	1.5	6	1000	4
18	2	1	3	1.2	8	1000	4
21	3	1	3	1.2	6	1000	8
23	4	1	3	1.5	6	1300	8
20	5	1	3	1.5	8	1300	4
19	6	1	3	1.2	6	1300	4
24	7	1	3	1.2	8	1300	8
22	8	1	3	1.5	8	1000	8
30	9	1	4	1.2	8	1000	8
32	10	1	4	1.5	8	1300	8
29	11	1	4	1.5	6	1000	8
31	12	1	4	1.2	6	1300	8
28	13	1	4	1.2	8	1300	4
26	14	1	4	1.5	8	1000	4
27	15	1	4	1.5	6	1300	4
25	16	1	4	1.2	6	1000	4
6	17	1	1	1.5	8	1000	8
8	18	1	1	1.2	8	1300	8
4	19	1	1	1.5	8	1300	4
7	20	1	1	1.5	6	1300	8
2	21	1	1	1.2	8	1000	4
1	22	1	1	1.5	6	1000	4
3	23	1	1	1.2	6	1300	4
5	24	1	1	1.2	6	1000	8
14	25	1	2	1.2	8	1000	8
9	26	1	2	1.2	6	1000	4
16	27	1	2	1.5	8	1300	8
11	28	1	2	1.5	6	1300	4
15	29	1	2	1.2	6	1300	8
12	30	1	2	1.2	8	1300	4
13	31	1	2	1.5	6	1000	8
10	32	1	2	1.5	8	1000	4

S2: Ablation Images for Blocks 2, 3, and 4 (Runs 1 to 32)

Block 3

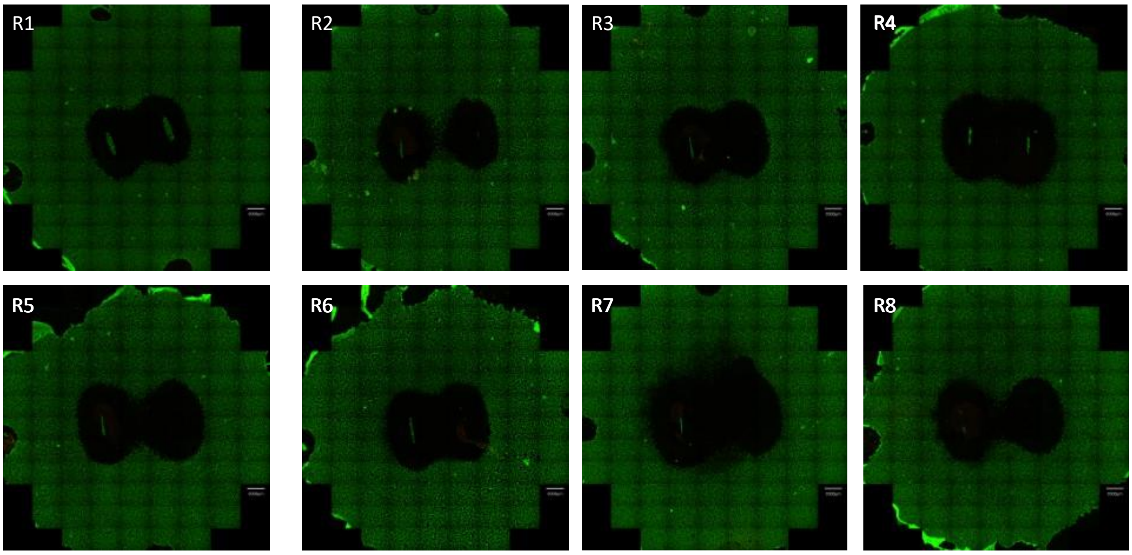

(a)

Block 4

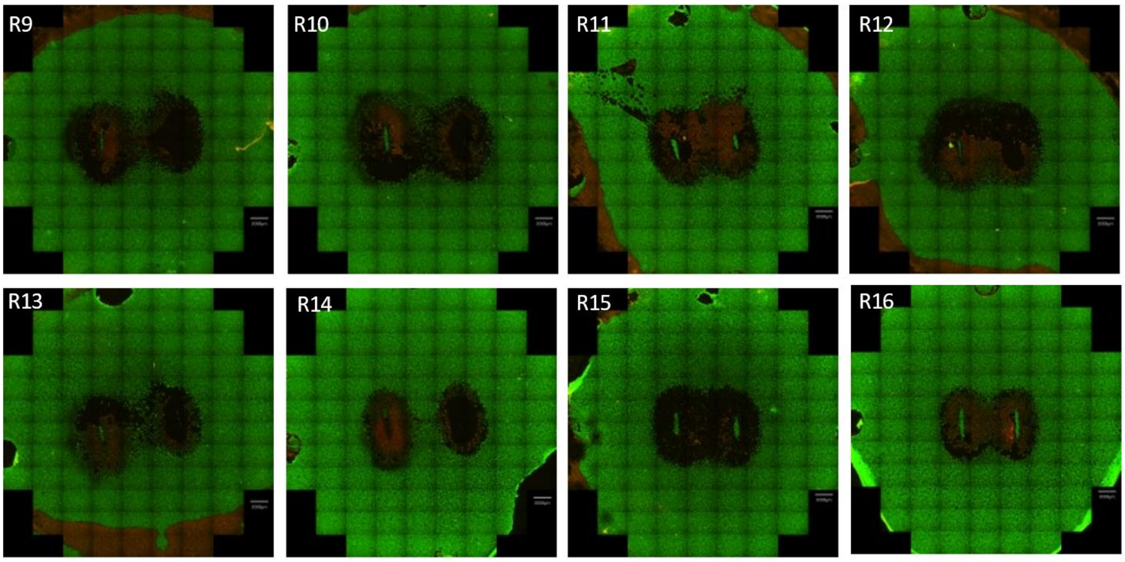

(b)

3lock 2

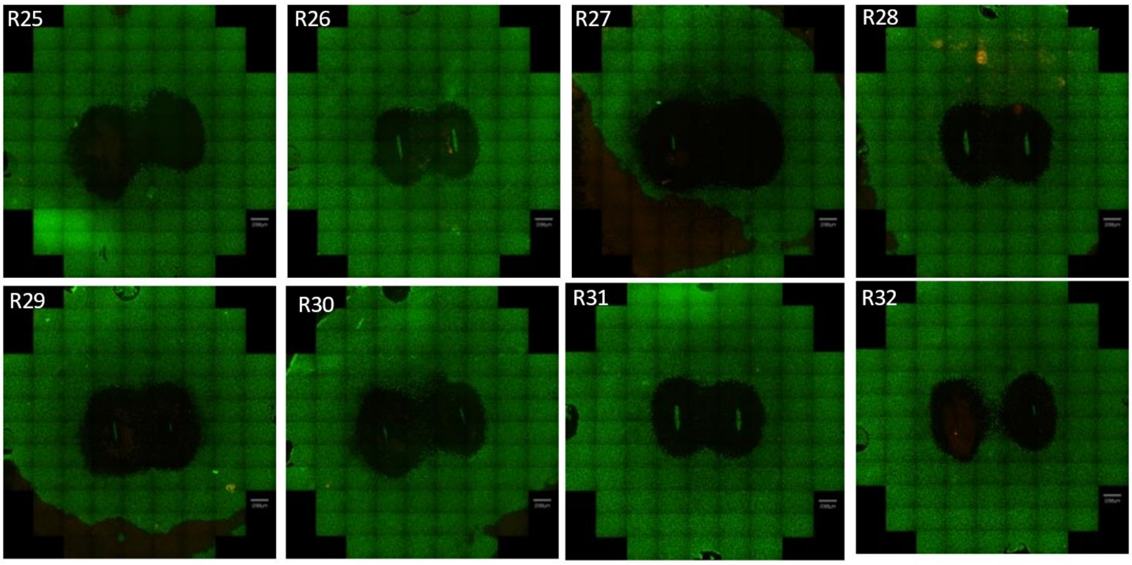

(c)

Figure S1: Ablated cells in well for each of the runs in Blocks 2, 3, and 4. Each image has the run number in the top right corner. The images are in order of ascending run number. Block 1 is presented in the paper. Grouping of images: (a) Block 3; (b) Block 4; (c) Block 2

S3: Measured Areas and Center Widths Per Run

Table S2: The Measured Areas and Centre Widths Per Run.

Run Order	Blocks	Electrode Width (mm)	Electrode Spacing c-c (mm)	Input Voltage (V)	Number of Ablation Repeats	Area (mm^2)	Center Width (mm)
1	3	1.5	6	1000	4	78.844	6.065
2	3	1.2	8	1000	4	82.621	1.179
3	3	1.2	6	1000	8	92.652	6.979
4	3	1.5	6	1300	8	115.289	9.003
5	3	1.5	8	1300	4	110.472	4.983
6	3	1.2	6	1300	4	93.139	7.469
7	3	1.2	8	1300	8	134.893	8.037
8	3	1.5	8	1000	8	108.61	4.53
9	4	1.2	8	1000	8	100.771	2.611
10	4	1.5	8	1300	8	118.351	6.251
11	4	1.5	6	1000	8	90.964	5.933
12	4	1.2	6	1300	8	93.343	6.702
13	4	1.2	8	1300	4	97.258	4.157
14	4	1.5	8	1000	4	70.369	0
15	4	1.5	6	1300	4	91.099	7.412
16	4	1.2	6	1000	4	73.082	5.135
17	1	1.5	8	1000	8	101.23	2.771
18	1	1.2	8	1300	8	128.282	6.909
19	1	1.5	8	1300	4	103.985	4.435
20	1	1.5	6	1300	8	107.71	8.472
21	1	1.2	8	1000	4	70.09	1.537
22	1	1.5	6	1000	4	63.428	5.016
23	1	1.2	6	1300	4	91.225	7.325
24	1	1.2	6	1000	8	86.142	5.504
25	2	1.2	8	1000	8	115.906	5.555
26	2	1.2	6	1000	4	88.626	6.624
27	2	1.5	8	1300	8	128.377	8.08
28	2	1.5	6	1300	4	92.227	7.418
29	2	1.2	6	1300	8	106.898	8.519
30	2	1.2	8	1300	4	104.998	6.008
31	2	1.5	6	1000	8	85.247	6.424
32	2	1.5	8	1000	4	82.644	1.212

S4: Minitab Regression Analysis for Ablation Area and Center Width

S4.1: Factorial Regression: Area versus Blocks, Electrode Width (mm), Electrode Spacing c-c (mm), Input Voltage (V), Num Ablation Repeats

Backward Elimination of Terms

α to remove = 0.1

Coded Coefficients

Term	Effect	Coef	SE Coef	T-Value	P-Value
Constant		97.149	0.919	105.68	0.000
Blocks					
1		-3.14	1.59	-1.97	0.061
2		3.47	1.59	2.18	0.040
3		4.92	1.59	3.09	0.005
Electrode Spacing c-c (mm)	13.059	6.529	0.919	7.10	0.000
Input Voltage (V)	20.395	10.198	0.919	11.09	0.000
Num Ablation Repeats	20.035	10.017	0.919	10.90	0.000
Electrode Spacing c-c (mm)*Input Voltage (V)	3.902	1.951	0.919	2.12	0.045
Electrode Spacing c-c (mm)*Num Ablation Repeats	6.713	3.357	0.919	3.65	0.001

Term	VIF
Constant	
Blocks	
1	1.50
2	1.50
3	1.50
Electrode Spacing c-c (mm)	1.00
Input Voltage (V)	1.00
Num Ablation Repeats	1.00
Electrode Spacing c-c (mm)*Input Voltage (V)	1.00
Electrode Spacing c-c (mm)*Num Ablation Repeats	1.00

Model Summary

S	R-sq	R-sq(adj)	R-sq(pred)
5.20040	93.52%	91.26%	87.45%

Analysis of Variance

Source	DF	Adj SS	Adj MS	F-Value	P-Value
Model	8	8973.6	1121.71	41.48	0.000
Blocks	3	588.2	196.08	7.25	0.001
Linear	3	7903.1	2634.36	97.41	0.000
Electrode Spacing c-c (mm)	1	1364.3	1364.27	50.45	0.000
Input Voltage (V)	1	3327.6	3327.65	123.04	0.000
Num Ablation Repeats	1	3211.2	3211.17	118.74	0.000
2-Way Interactions	2	482.3	241.16	8.92	0.001
Electrode Spacing c-c (mm)*Input Voltage (V)	1	121.8	121.80	4.50	0.045
Electrode Spacing c-c (mm)*Num Ablation Repeats	1	360.5	360.51	13.33	0.001
Error	23	622.0	27.04		
Total	31	9595.7			

Regression Equation in Uncoded Units

Area = 118.4 - 18.50 Electrode Spacing c-c (mm) - 0.0231 Input Voltage (V)
- 6.74 Num Ablation Repeats + 0.01301 Electrode Spacing c-c (mm)*Input Voltage (V)
+ 1.678 Electrode Spacing c-c (mm)*Num Ablation Repeats

Equation averaged over blocks.

Fits and Diagnostics for Unusual Observations

Obs	Area	Fit	Resid	Std Resid
22	63.43	72.57	-9.15	-2.07 R
26	88.63	79.18	9.45	2.14 R

R Large residual

Alias Structure

Factor	Name
A	Electrode Width (mm)
B	Electrode Spacing c-c (mm)
C	Input Voltage (V)
D	Num Ablation Repeats

Aliases
I
Block 1 - ABCD
Block 2 + ABCD
Block 3 - ABCD
B
C
D
BC
BD

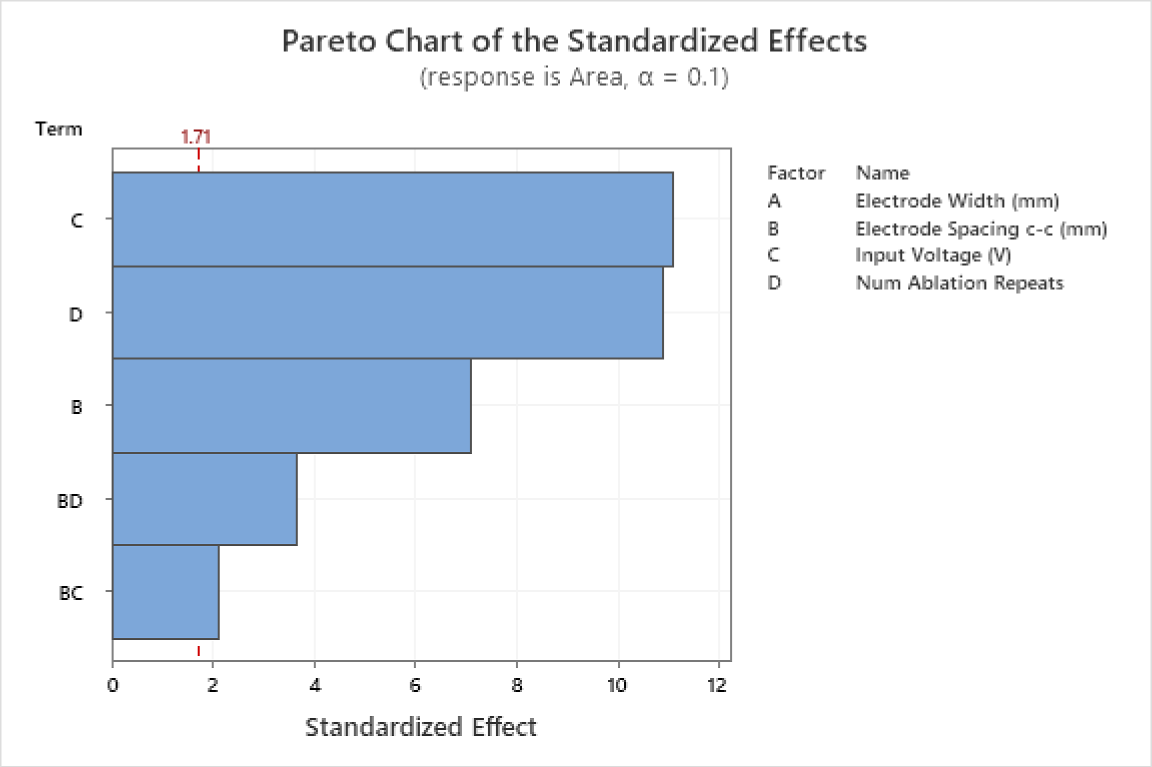
Pareto Chart of the Standardized Effects
(response is Area, α = 0.1)
Term
1.71
C
D
B
BD
BC
0
2
4
6
8
10
12
Standardized Effect
Factor Name
A Electrode Width (mm)
B Electrode Spacing c-c (mm)
C Input Voltage (V)
D Num Ablation Repeats

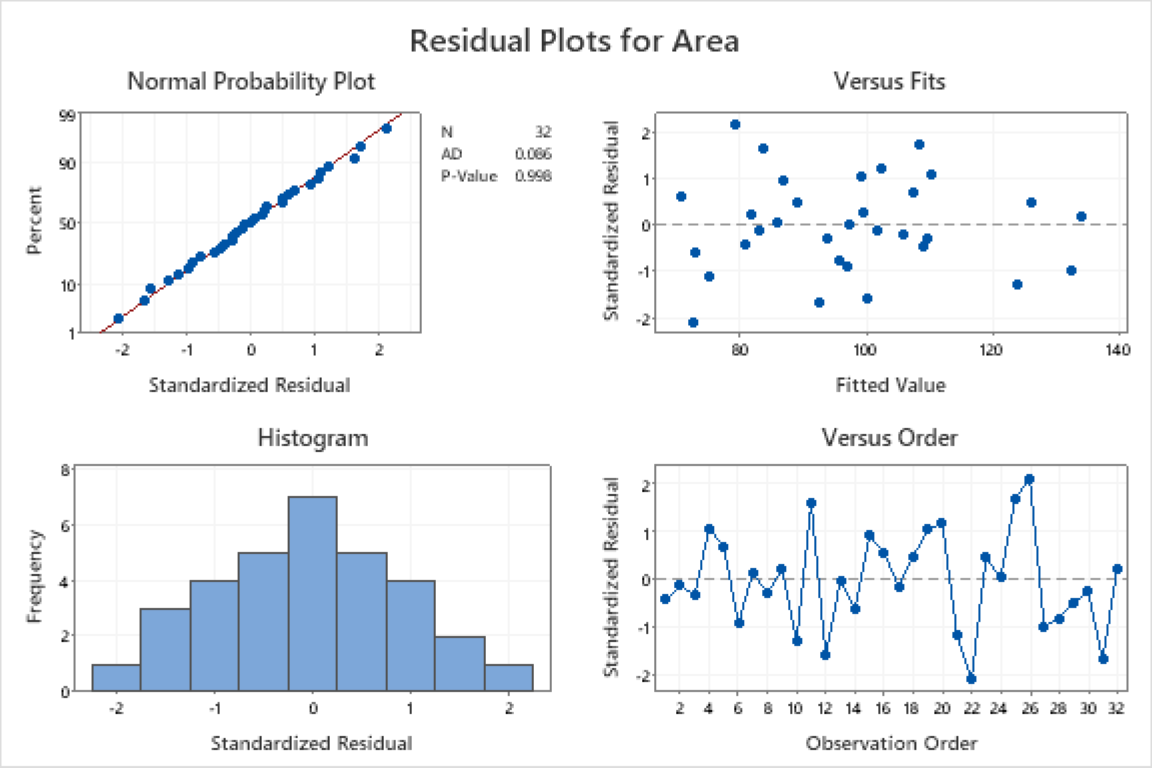
Residual Plots for Area
Normal Probability Plot
Percent
Standardized Residual
N 32
AD 0.086
P-Value 0.998
Versus Fits
Standardized Residual
Fitted Value
Histogram
Frequency
Standardized Residual
Versus Order
Standardized Residual
Observation Order

S4.2: Factorial Regression: Centerwidth (manual) versus Blocks, Electrode Width (mm), Electrode Spacing c-c (mm), Input Voltage (V), Num Ablation Repeats

Backward Elimination of Terms

α to remove = 0.1

Coded Coefficients

Term	Effect	Coef	SE Coef	T-Value	P-Value
Constant		5.5705	0.0983	56.70	0.000
Blocks					
1		-0.324	0.170	-1.91	0.069
2		0.660	0.170	3.88	0.001
3		0.460	0.170	2.70	0.013
Electrode Spacing c-c (mm)	-2.6091	-1.3045	0.0983	-13.28	0.000
Input Voltage (V)	2.7566	1.3783	0.0983	14.03	0.000
Num Ablation Repeats	1.6441	0.8220	0.0983	8.37	0.000
Electrode Spacing c-c (mm)*Input Voltage (V)	0.9266	0.4633	0.0983	4.72	0.000
Electrode Spacing c-c (mm)*Num Ablation Repeats	1.0101	0.5050	0.0983	5.14	0.000

Term	VIF
Constant	
Blocks	
1	1.50
2	1.50
3	1.50
Electrode Spacing c-c (mm)	1.00
Input Voltage (V)	1.00
Num Ablation Repeats	1.00
Electrode Spacing c-c (mm)*Input Voltage (V)	1.00
Electrode Spacing c-c (mm)*Num Ablation Repeats	1.00

Model Summary

S	R-sq	R-sq(adj)	R-sq(pred)
0.555800	95.82%	94.37%	91.91%

Analysis of Variance

Source	DF	Adj SS	Adj MS	F-Value	P-Value
Model	8	162.976	20.3720	65.95	0.000
Blocks	3	11.076	3.6920	11.95	0.000
Linear	3	136.870	45.6234	147.69	0.000
Electrode Spacing c-c (mm)	1	54.458	54.4577	176.29	0.000
Input Voltage (V)	1	60.789	60.7891	196.78	0.000
Num Ablation Repeats	1	21.624	21.6235	70.00	0.000
2-Way Interactions	2	15.030	7.5150	24.33	0.000
Electrode Spacing c-c (mm)*Input Voltage (V)	1	6.868	6.8681	22.23	0.000
Electrode Spacing c-c (mm)*Num Ablation Repeats	1	8.162	8.1618	26.42	0.000
Error	23	7.105	0.3089		
Total	31	170.081			

Regression Equation in Uncoded Units

Centrewidth (manual) = 37.14 - 6.371 Electrode Spacing c-c (mm)
- 0.01243 Input Voltage (V)
- 1.357 Num Ablation Repeats
+ 0.003089 Electrode Spacing c-c (mm)*Input Voltage (V)
+ 0.2525 Electrode Spacing c-c (mm)*Num Ablation Repeats

Equation averaged over blocks.

Fits and Diagnostics for Unusual Observations

Obs	Centrewidth (manual)	Fit	Resid	Std Resid
25	5.555	4.411	1.144	2.43 R

R Large residual

Alias Structure

Factor	Name
A	Electrode Width (mm)
B	Electrode Spacing c-c (mm)
C	Input Voltage (V)
D	Num Ablation Repeats

Aliases
I
Block 1 - ABCD
Block 2 + ABCD
Block 3 - ABCD
B
C
D
BC
BD

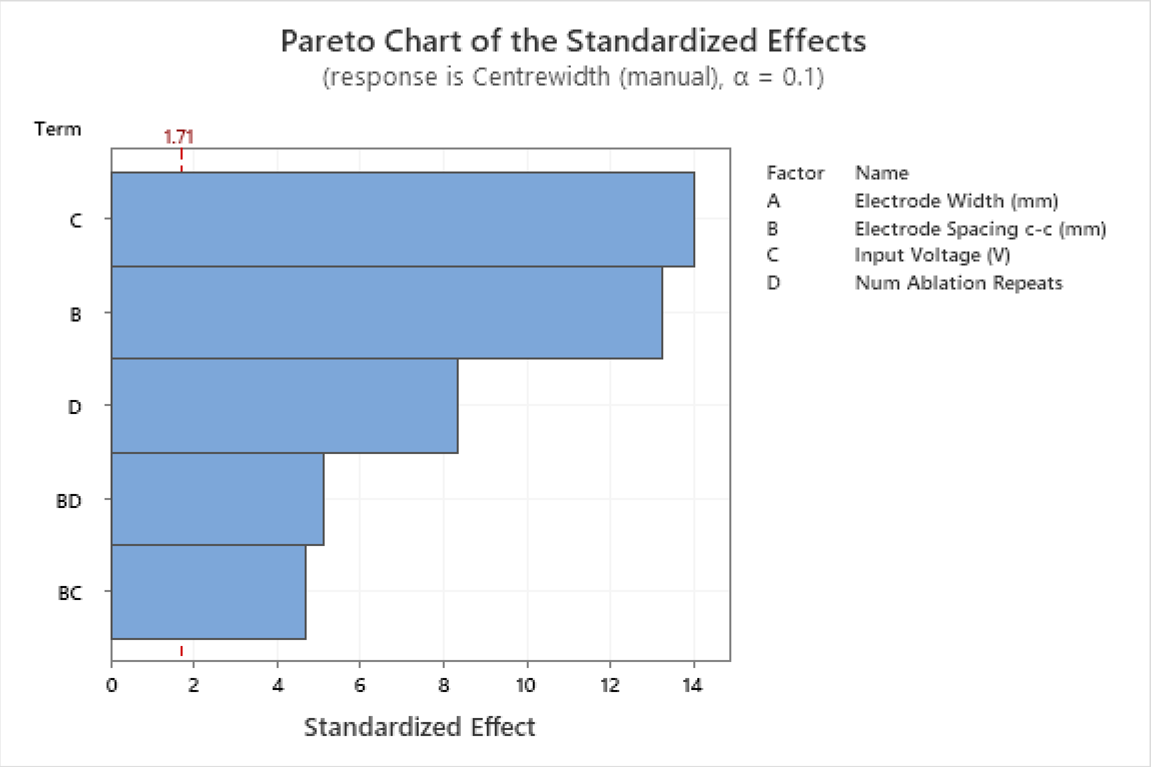
Pareto Chart of the Standardized Effects
(response is Centrewidth (manual), α = 0.1)
Term
1.71
C
B
D
BD
BC
0
2
4
6
8
10
12
14
Standardized Effect
Factor Name
A Electrode Width (mm)
B Electrode Spacing c-c (mm)
C Input Voltage (V)
D Num Ablation Repeats

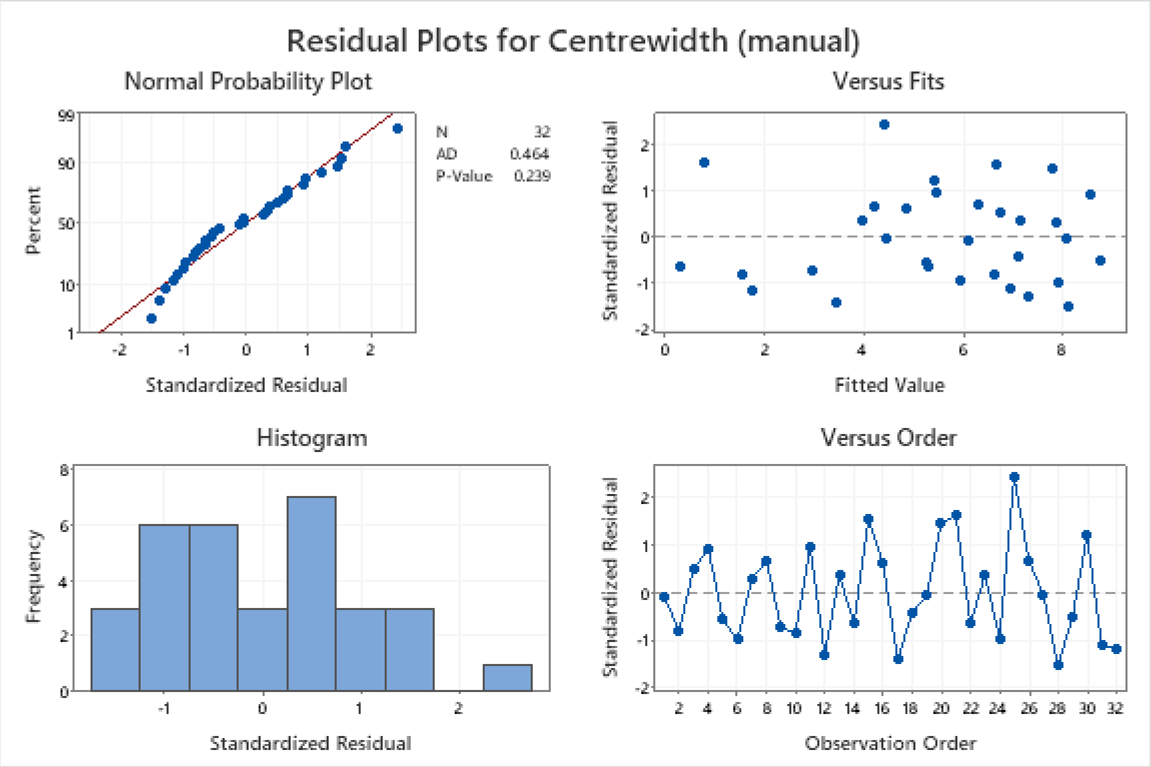
Residual Plots for Centrewidth (manual)
Normal Probability Plot
Percent
Standardized Residual
N 32
AD 0.464
P-Value 0.239
Versus Fits
Standardized Residual
Fitted Value
Histogram
Frequency
Standardized Residual
Versus Order
Standardized Residual
Observation Order

S5: Confirmation Run Optimisation – Minitab Analysis

S5.1. Response Optimization: Area 97 mm2 1000 V

Parameters

Response	Goal	Lower	Target	Upper	Weight	Importance
Area	Target	63.428	97	134.893	1	1

Variable Ranges

Variable	Values
Electrode Spacing c-c (mm)	8
Input Voltage (V)	1000
Num Ablation Repeats	(4, 8)

Solution

Solution	Electrode Spacing c-c (mm)	Input Voltage (V)	Num Ablation Repeats	Area Fit	Composite Desirability
1	8	1000	6.81799	97	1

Multiple Response Prediction

Variable	Setting
Electrode Spacing c-c (mm)	8
Input Voltage (V)	1000
Num Ablation Repeats	6.81799

Response	Fit	SE Fit	95% CI	95% PI
Area	97.00	1.91	(93.04, 100.96)	(85.54, 108.46)

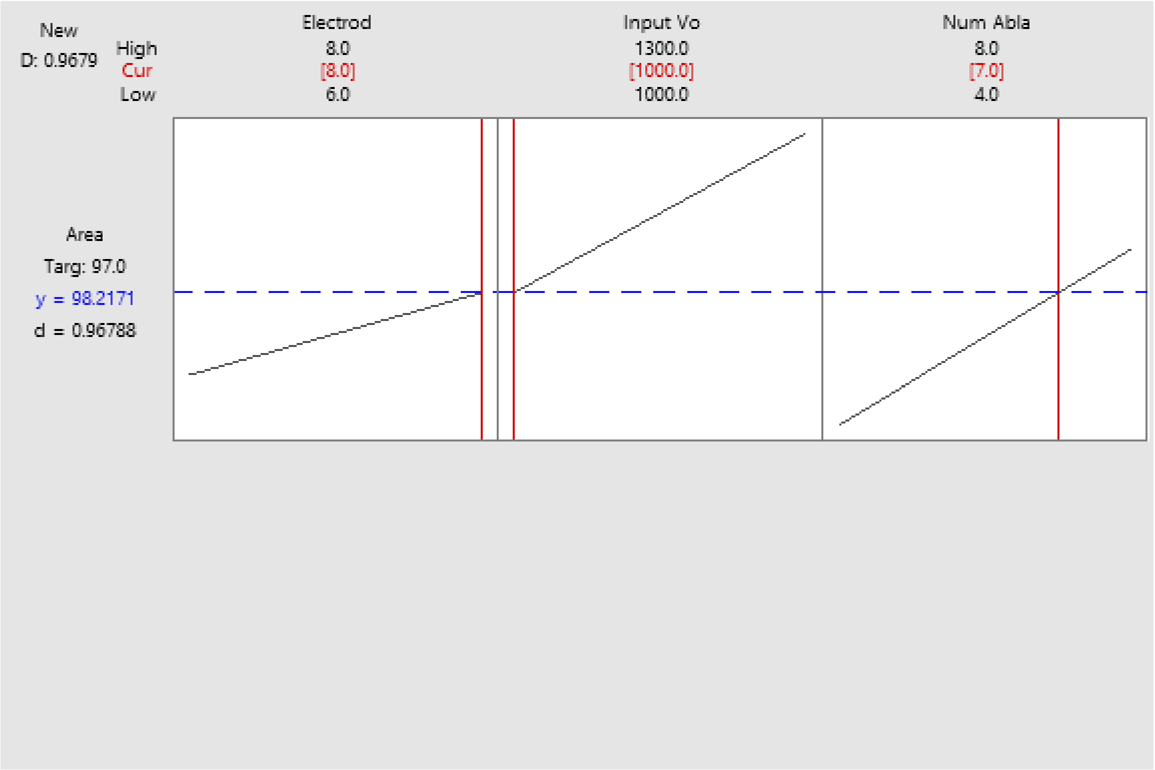

S5.2. Response Optimization: Area 97 mm2 1300 V

Parameters

Response	Goal	Lower	Target	Upper	Weight	Importance
Area	Target	63.428	97	134.893	1	1

Variable Ranges

Variable	Values
Electrode Spacing c-c (mm)	8
Input Voltage (V)	1300
Num Ablation Repeats	(4, 8)

Solution

Solution	Electrode Spacing c-c (mm)	Input Voltage (V)	Num Ablation Repeats	Area Fit	Composite Desirability
1	8	1300	4	102.453	0.856093

Multiple Response Prediction

Variable	Setting
Electrode Spacing c-c (mm)	8
Input Voltage (V)	1300
Num Ablation Repeats	4

Response	Fit	SE Fit	95% CI	95% PI
Area	102.45	2.25	(97.79, 107.11)	(90.73, 114.18)

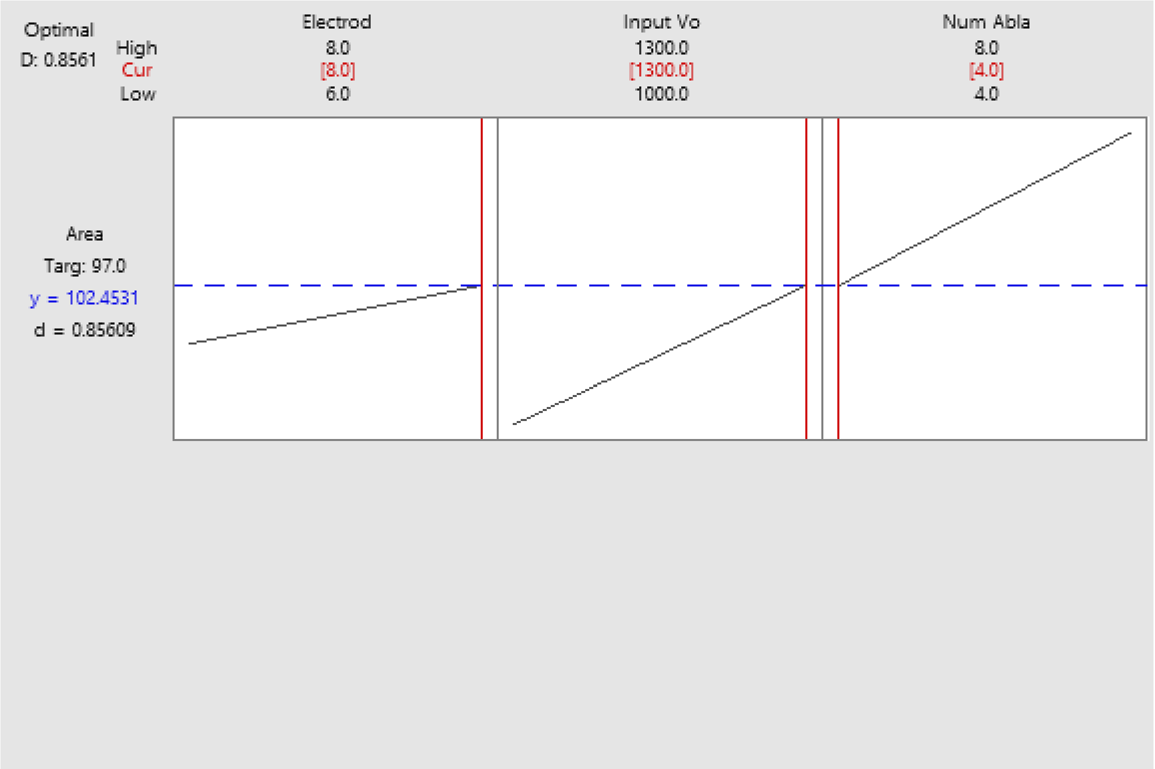

S5.3: Response Optimization: Area 80 mm2

Parameters

Response	Goal	Lower	Target	Upper	Weight	Importance
Area	Target	63.428	80	134.893	1	1

Variable Ranges

Variable	Values
Electrode Spacing c-c (mm)	8
Input Voltage (V)	(1000, 1300)
Num Ablation Repeats	(4, 8)

Solution

Solution	Electrode Spacing c-c (mm)	Input Voltage (V)	Num Ablation Repeats	Area Fit	Composite Desirability
1	8	1000	4.27573	80	1

Multiple Response Prediction

Variable	Setting
Electrode Spacing c-c (mm)	8
Input Voltage (V)	1000
Num Ablation Repeats	4.27573

Response	Fit	SE Fit	95% CI	95% PI
Area	80.00	2.15	(75.55, 84.45)	(68.36, 91.64)

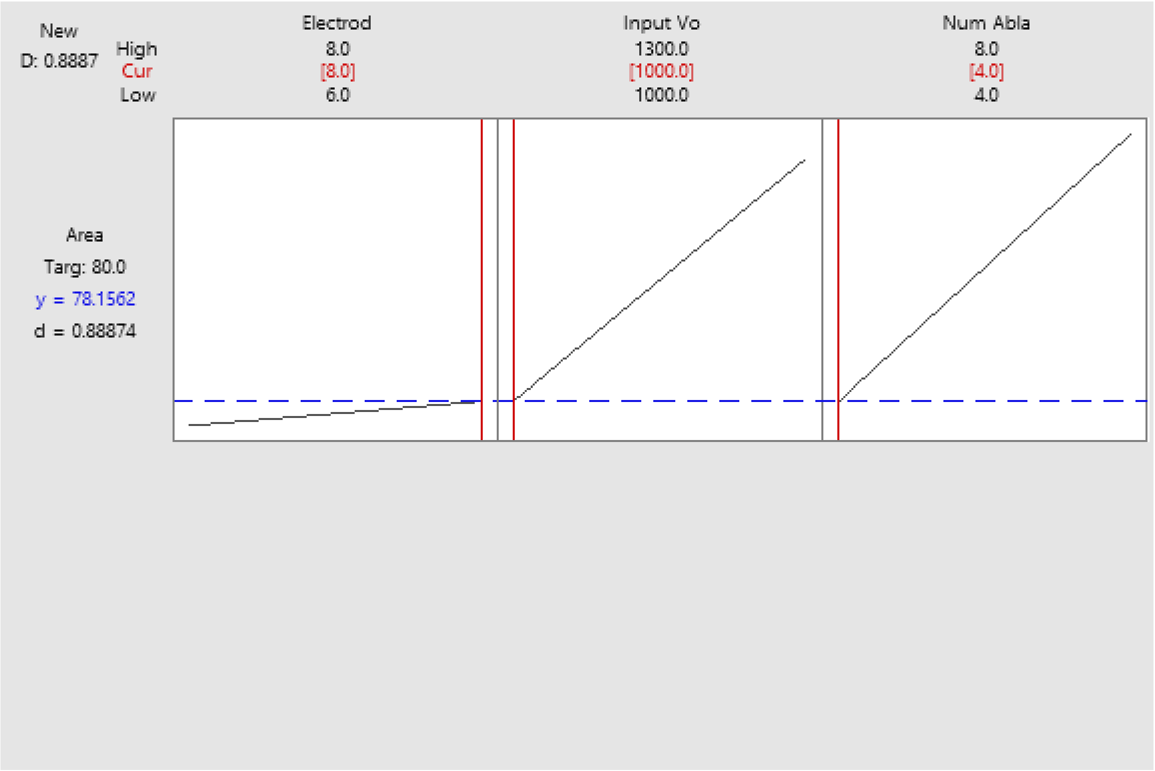

S5.4: Response Optimization: Area 110 mm2

Parameters

Response	Goal	Lower	Target	Upper	Weight	Importance
Area	Target	63.428	110	134.893	1	1

Variable Ranges

Variable	Values
Electrode Spacing c-c (mm)	8
Input Voltage (V)	(1000, 1300)
Num Ablation Repeats	(4, 8)

Solution

Solution	Electrode Spacing c-c (mm)	Input Voltage (V)	Num Ablation Repeats	Area Fit	Composite Desirability
1	8	1150	6.94534	110	1

Multiple Response Prediction

Variable	Setting
Electrode Spacing c-c (mm)	8
Input Voltage (V)	1150
Num Ablation Repeats	6.94534

Response	Fit	SE Fit	95% CI	95% PI
Area	110.00	1.44	(107.03, 112.97)	(98.84, 121.16)

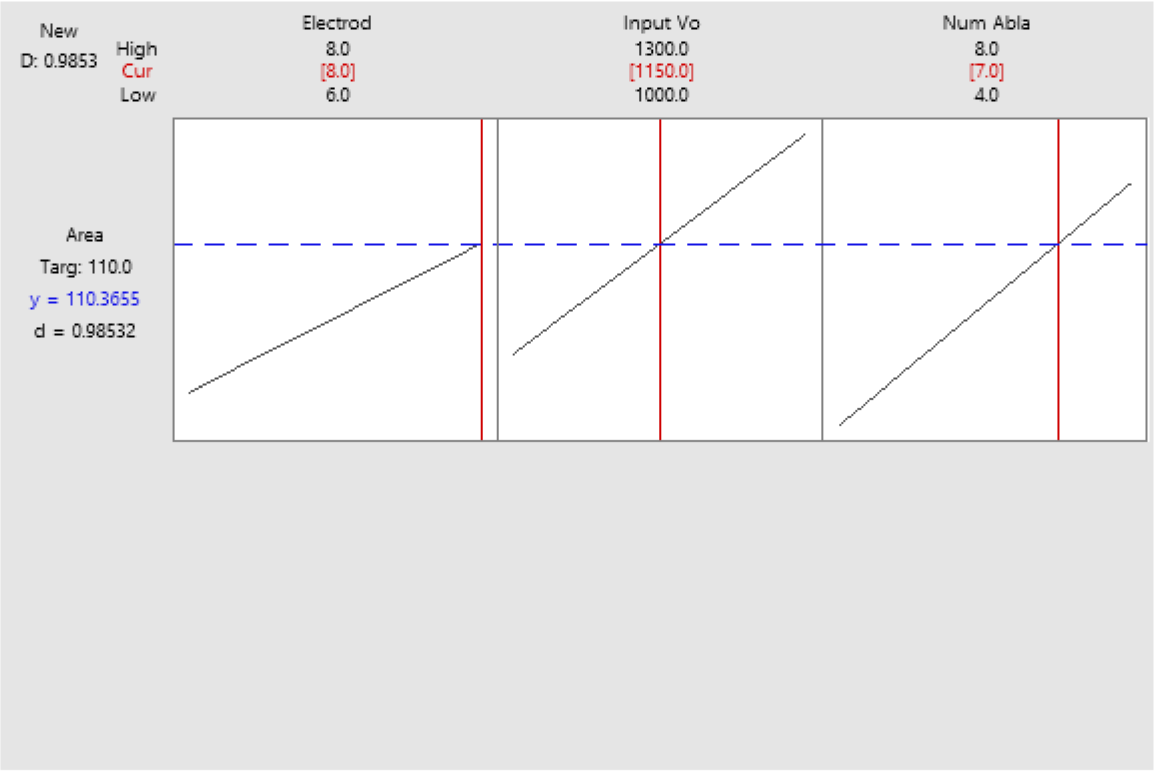

S6: Confirmation Run Analysis (Prediction for Area @ 98.75%) – Minitab Analysis

Regression Equation in Uncoded Units

Area = 118.4 - 18.50 Electrode Spacing c-c (mm) - 0.0231 Input Voltage (V)
- 6.74 Num
Ablation Repeats + 0.01301 Electrode Spacing c-c (mm)*Input Voltage (V)
+ 1.678 Electrode Spacing c-c (mm)*Num Ablation Repeats

Equation averaged over blocks.

Settings

Variable	Setting
Electrode Spacing c-c (mm)	8
Input Voltage (V)	1000
Num Ablation Repeats	7

Prediction is averaged over blocks.

Prediction

Fit	SE Fit	98.75% CI	98.75% PI
98.2171	1.95015	(92.9327, 103.501)	(83.1672, 113.267)

Settings

Variable	Setting
Electrode Spacing c-c (mm)	8
Input Voltage (V)	1300
Num Ablation Repeats	4

Prediction is averaged over blocks.

Prediction

Fit	SE Fit	98.75% CI	98.75% PI
102.453	2.25184	(96.3512, 108.555)	(87.0971, 117.809)

Settings

Variable	Setting
Electrode Spacing c-c (mm)	8
Input Voltage (V)	1000
Num Ablation Repeats	4

Prediction is averaged over blocks.

Prediction

Fit	SE Fit	98.75% CI	98.75% PI
78.1562	2.25184	(72.0543, 84.2580)	(62.8002, 93.5122)

Settings

Variable	Setting

Electrode Spacing c-c (mm)	8
Input Voltage (V)	1150
Num Ablation Repeats	7

Prediction is averaged over blocks.

Prediction

Fit	SE Fit	98.75% CI	98.75% PI
110.366	1.45356	(106.427, 114.304)	(95.7338, 124.997)

S7: Electrical Isolation Optimisation – Minitab Analysis

Parameters

Response	Goal	Lower	Target	Upper	Weight	Importance
Centrewidth (manual)	Target	0.000	4.500	9.003	1	1
Area	Maximum	63.428	134.893		1	1

Variable Ranges

Variable	Values
Electrode Spacing c-c (mm)	8
Input Voltage (V)	(1000, 1300)
Num Ablation Repeats	(4, 8)

Solution

Solution	Electrode Spacing c-c (mm)	Input Voltage (V)	Num Ablation Repeats	Centrewidth (manual) Fit	Area Fit	Composite Desirability
1	8	1060.97	8	4.5	109.842	0.805895

Multiple Response Prediction

Variable	Setting
Electrode Spacing c-c (mm)	8
Input Voltage (V)	1060.97
Num Ablation Repeats	8

Response	Fit	SE Fit	95% CI	95% PI
Centrewidth (manual)	4.500	0.213	(4.059, 4.941)	(3.269, 5.731)
Area	109.84	1.99	(105.72, 113.97)	(98.32, 121.36)

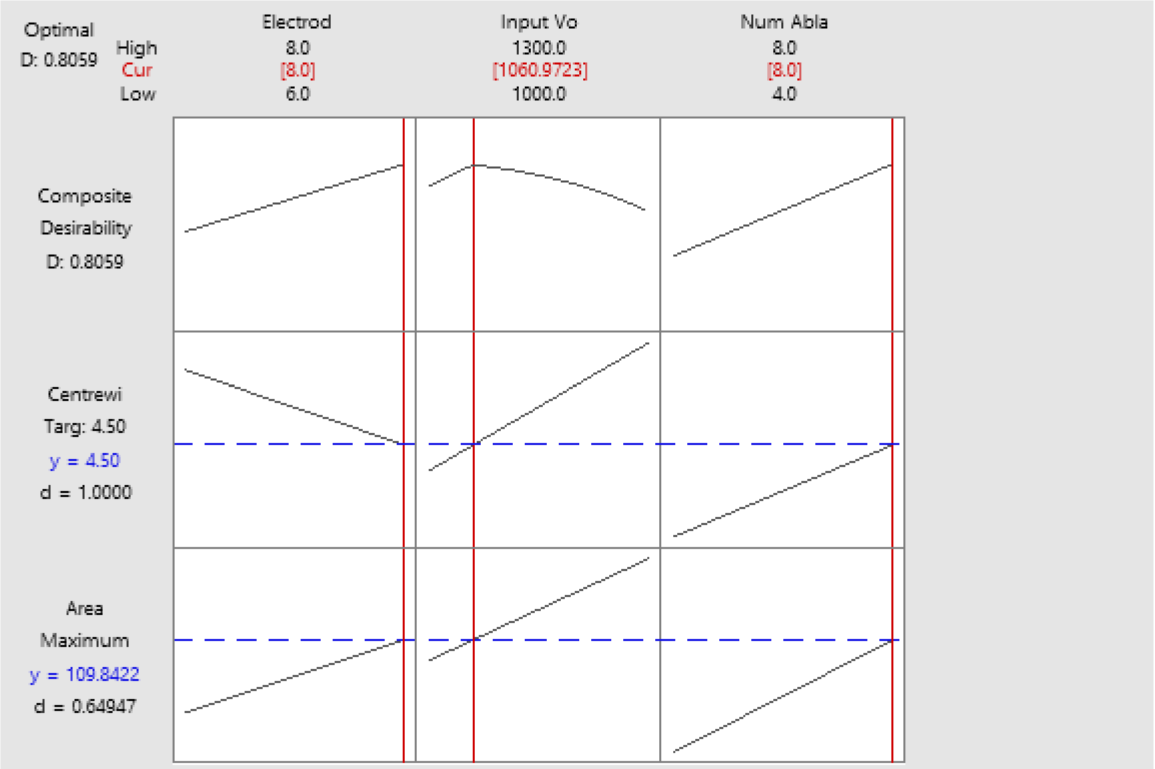
Optimal
D: 0.8059
High
Cur
Low
Electrod
8.0
[8.0]
6.0
Input Vo
1300.0
[1060.9723]
1000.0
Num Abla
8.0
[8.0]
4.0
Composite
Desirability
D: 0.8059
Centrewi
Targ: 4.50
y = 4.50
d = 1.0000
Area
Maximum
y = 109.8422
d = 0.64947
